# A new perspective on binaural integration using response time methodology: super capacity revealed in conditions of binaural masking release

**DOI:** 10.3389/fnhum.2014.00641

**Published:** 2014-08-22

**Authors:** Jennifer J. Lentz, Yuan He, James T. Townsend

**Affiliations:** ^1^Department of Speech and Hearing Sciences, Indiana UniversityBloomington, IN, USA; ^2^Department of Psychological and Brain Sciences, Indiana UniversityBloomington, IN, USA

**Keywords:** reaction time, binaural hearing, masking release, systems factorial technology, workload capacity

## Abstract

This study applied reaction-time based methods to assess the workload capacity of binaural integration by comparing reaction time (RT) distributions for monaural and binaural tone-in-noise detection tasks. In the diotic contexts, an identical tone + noise stimulus was presented to each ear. In the dichotic contexts, an identical noise was presented to each ear, but the tone was presented to one of the ears 180° out of phase with respect to the other ear. Accuracy-based measurements have demonstrated a much lower signal detection threshold for the dichotic vs. the diotic conditions, but accuracy-based techniques do not allow for assessment of system dynamics or resource allocation across time. Further, RTs allow comparisons between these conditions at the same signal-to-noise ratio. Here, we apply a reaction-time based capacity coefficient, which provides an index of workload efficiency and quantifies the resource allocations for single ear vs. two ear presentations. We demonstrate that the release from masking generated by the addition of an identical stimulus to one ear is limited-to-unlimited capacity (efficiency typically less than 1), consistent with less gain than would be expected by probability summation. However, the dichotic presentation leads to a significant increase in workload capacity (increased efficiency)—most specifically at lower signal-to-noise ratios. These experimental results provide further evidence that configural processing plays a critical role in binaural masking release, and that these mechanisms may operate more strongly when the signal stimulus is difficult to detect, albeit still with nearly 100% accuracy.

## Introduction

An integral question in psychoacoustics is that of binaural integration: how information presented to the two ears is combined in order to form a unified percept. In natural environments, the sounds received by the two ears are typically different from one another, but experiments using headphones allow identical stimuli to be presented to both ears. It is well-known that identical auditory stimuli presented to each ear are perceived as a single sound (e.g., Leakey et al., 1958), but there are also many instances in which unified percepts are elicited when different signals are presented to the two ears (e.g., if a sound source is presented to one side of a listener). In his seminal work on the “cocktail party effect,” Cherry (1953) demonstrated that the auditory system generates fused percepts of auditory sources in sophisticated listening situations. Although multiple cues are used by the auditory system to accomplish this goal, the binaural system is a critical component of this process (see Bregman, 1994 for a review).

One notable aspect of many studies is that they evaluate the mechanisms responsible for detection using threshold- and accuracy-based techniques. Accuracy based methods can answer many important questions pertaining to various aspects of perception and cognition. Yet, they are inherently limited when issues pertaining to dynamic mechanisms are raised, since by definition they ignore temporal features of the system and correlate data (e.g., see Van Zandt and Townsend, 2013).

We can apply a separate strain of research in perceptual and cognitive psychology which focuses on multiple signals vs. a single signal (or more specifically, two ears vs. one ear) and primarily uses reaction time (RT) for its dependent variable. We will refer to that approach as the “redundant signals approach” (cf. Bernstein, 1970; Grice et al., 1984). Its terminology is, of course, rather different than that typically employed in the hearing domain but we will strive to provide sufficient bridges across the divide.

Within that general domain, strong tools have been developed that can assist the investigator in unveiling the dynamics of the underlying perceptual system. We suggest that the two basic measures, accuracy, and RT, can together go a long way in answering fundamental questions within binaural hearing. In fact, statistics derived within a theoretical, information processing framework have led to theory-driven methodologies within which various aspects of cognitive sensory processing can be evaluated.

The fundamental goal of this study is to apply the redundant signals techniques to further our understanding of the mechanisms responsible for integrating information across the ears. However, we need to first review some of the germane, basic findings in the binaural literature. Almost all of these were accuracy based but a few measured RTs.

Several psychophysical approaches have been taken to address the fundamental question of binaural integration with a substantial proportion of experiments using a basic task—detecting a tone added to a band of noise. In these experiments, the detection threshold level of the tone is typically measured (cf. Fletcher, 1940). The tone + noise stimulus can be presented to a single ear, commonly referred to as *monaural* presentation, denoted *N_m_S_m_*, where *N* refers to the noise, *S* refers to the tonal signal, and *m* denotes the monaural presentation. The tone + noise stimulus can also be presented to both ears. If both ears receive identical signals, we refer to this as a *diotic*, homophasic presentation, *N*_0_*S*_0_, where 0 represents identical noise (*N*_0_) and identical tone (*S*_0_) presented to each ear. A number of psychophysical studies have demonstrated that presenting a tone-in-noise diotically yields, at most, a marginal improvement in the detection threshold of the pure tone compared to a monaural presentation (e.g., Hirsh and Burgeat, 1958; Egan et al., 1969; Davidson et al., 2006).

In fact, to date, thresholds for *N*_0_*S*_0_ and *N_m_S_m_* are generally treated as being the same (cf. Durlach and Colburn, 1978). For threshold-based tests, then, there appears to be little or no benefit to having the redundant tone-in-noise presented to a second ear, although a small benefit has been reported for detecting pure tones in quiet (cf. Moore, 2013). Consequently, performance in the diotic conditions (for tones alone or tones in noise) is worse than a probability summation model would predict with accuracy being, at best, slightly better for two ears compared to one.

Of course, natural conditions typically allow the two ears to receive different signals. Such a situation would occur when a sound source is not directly in front of the listener. Any instance in which the ears receive different signals is referred to as *dichotic* listening. In a very special case, when presenting sounds over headphones, one can present a noise source identical (correlated) between ears (*N*_0_) with a signal source uncorrelated between the ears. If the signal stimulus is presented π radians out of phase across the ears, we refer to this as an antiphasic presentation, *N*_0_*S*_π_. Here, the signal level at threshold is much lower than in the *N*_0_*S*_0_ condition, with the difference in threshold commonly referred to as the binaural masking level difference (BMLD; e.g., Hirsh, 1948; Jeffress et al., 1952; Egan, 1965; Henning, 1965; Henning et al., 2005; Davidson et al., 2009). The dichotic stimulation thus leads to superior accuracy over either monaural or diotic performance. Models of these types of psychophysical data include processes of interaural cross-correlation, equalization and cancelation, and across-ear inhibition (e.g., Bernstein et al., 1999; Breebaart et al., 2001; Davidson et al., 2009).

To summarize, first the performance in the diotic conditions is worse than a probability summation model would predict but with a slightly better relative accuracy in the binaural vs. monaural conditions. Secondly, dichotic stimulation with inverted tones leads to superior performance. An ideal detector which could cancel the noise would allow for this superior result, but would predict signal detection thresholds in *N*_0_*S*_π_ to be the same as in quiet (Durlach and Colburn, 1978). Because masking still does occur (that is, thresholds in *N*_0_*S*_π_ are not equivalent to unmasked thresholds), the noise cancelation process, though robust, is imperfect.

Both these findings indicate the absence of independent detection with each detector being the same (i.e., just as good but no better) with both ears functioning as with only one. The substandard performance in the diotic conditions could presumably be due to limitations in capacity (i.e., caused by inadequate resources available to both ears simultaneously or perhaps to mutual channel inhibition). However, the superior performance found with the dichotic conditions suggests, as noted, some type of either energy or activation summation or, contrarily, a type of information interaction as intimated by the cross-correlation interpretation.

Moving on to consider what has been accomplished in the binaural detection domain with RT as the dependent variable, in 1944, Chocholle was the first to measure RTs for binaural vs. monaural stimulation, demonstrating that binaural detection of pure tones (in quiet) was faster than monaural detection. Simon (1967) showed that the difference in mean RT between binaural and monaural stimulation was very small (about 4 ms for an average 200 ms RT) but statistically significant. More recently, Schlittenlacher et al. (2014) also demonstrated a 5–10 ms binaural advantage in RT. These studies reported only mean RTs and without a deeper quantitative analysis, one is challenged to establish how activation of the two ears relates to resource allocation.

A seminal RT based study within the domain of redundant signals literature, was undertaken by Schröter et al. (2007) who reported RT distributions for detection of a 300-ms, 60 dB SPL pure tone presented to the left ear, the right ear, or both ears. Whether the two tones had identical or different frequencies, there was little evidence for a redundant-signal benefit. That is, although RTs were slightly faster for detecting two tones vs. one tone, the increase in RT was less than would be expected under probability summation. However, in a second experiment, one of the tones was replaced by a noise, and here they found faster RTs than would be predicted by a probability summation model. We will discuss the Schröter et al. (2007) results alongside our own.

Our approach here will be to implement a suite of tools from the theory-driven RT methodology, “systems factorial technology” (subsequently SFT) originated by Townsend and colleagues (e.g., Townsend and Nozawa, 1995; Townsend and Wenger, 2004a). This methodology permits the simultaneous assessment of a number of critical information processing mechanisms within the same experimental paradigm. These tools will allow an analysis of resource allocation and interaction between the two ears and also provides for psychophysical assessment under very different conditions than accuracy- or threshold-based measures.

First, RTs can be measured under conditions of very high accuracy, tapping into different locations on the psychometric function. With respect to BMLD studies, the psychometric functions for detecting a tone added to noise in the *N*_0_*S*_0_ and *N*_0_*S*_π_ contexts are parallel but they do not overlap when the masking release is large (Egan et al., 1969). Because the psychometric functions do not overlap, auditory mechanisms are evaluated for these two contexts at largely different SNRs. Given the nonlinear nature of the ear, it is indeed possible that different auditory mechanisms may be invoked at the two different SNRs estimated at threshold. Second, accuracy-based techniques do not allow easy assessment of the dynamics of the system without clever stimulus manipulations that can be difficult to implement acoustically. Finally, RT measures can provide a complement to accuracy-based measures in our attempt at converging on a unified understanding of the mechanisms responsible for perception. Since the broad suite of tools available in SFT has not heretofore been implemented in binaural perception and not at all to the release from masking phenomenon, the following section provides a brief tutorial.

## Architecture: the serial vs. parallel issue

One of the first issues to address is the form, or the architecture, used by a system. We define *serial processing* as processing things one at a time or sequentially, with no overlap among the successive processing times. Processing might mean search for a target among a set of distractors in memory or in a display, solving facets of a problem, deciding among a set of objects, and so on. *Parallel processing* means processing all things simultaneously, although it is allowed that each process may finish at different times (Townsend et al., 2011).

**Figure 1:**
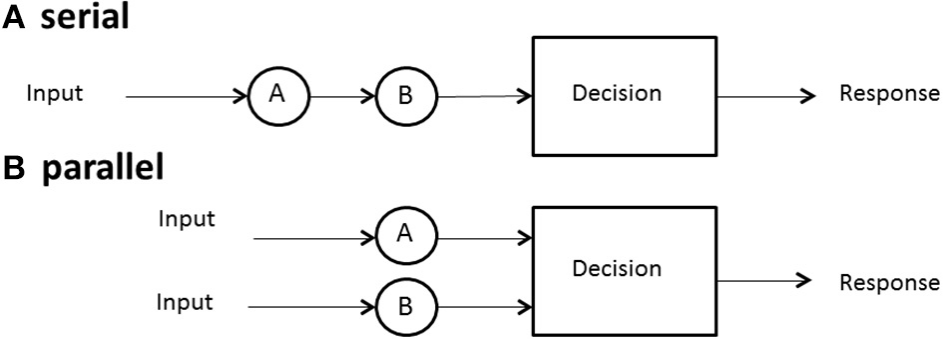
**Depiction of two systems: (A) serial and (B) parallel**.

Although the term *architecture* might seem to imply rigid structure, we may also employ it to refer to more flexible arrangements. Thus, it might be asserted that certain neural systems are, at least by adulthood, fairly wired in and that they act in parallel (or in some cases, in serial). On the other hand, a person might scan the newspaper for, say, two terms, one at a time, that, is serially or, by dint of will, might try to scan for them in parallel. Although parallel vs. serial processing is in some sense the most elemental pair of architectures, much more complexity can be imagined and, indeed, investigated theoretically and empirically (e.g., Schweickert, 1978; Schweickert and Townsend, 1989). Figure 1 illustrates the architecture associated with serial and parallel processing.

If we are dealing with only one or two channels or items, we shall often just refer to these as *a* or *b*, but if we must consider the general case of arbitrary *n* items or channels, we list them as 1, 2,…, *n* − 1, *n*. In a serial system, then, if *n* = 2, and channels *a* and *b* are stochastically independent (see subsequent material for more on this issue), then the density of the sum of the two serial times is the convolution of the separate densities (Townsend and Ashby, 1983, p. 30).

This new density is designated as *f_a_*(*t*) ^*^ *f_b_*(*t*), where the asterisk denotes convolution and *a* and *b* are processed serially. The mean or expectation of the sum *E*[*T_a_* + *T_b_*] = *E*[*T_a_*] + *E*[*T_b_*] indicates that the overall completion time for serial processes is the sum of all the individual means. The standard serial model requires that *f_a_*(*t*) = *f_b_*(*t*), which in turn implies that *E*[*T_a_*] = *E*[*T_b_*] = *E*[*T*], and *E*[*T_a_* + *T_b_*] = 2*E*[*T*].

In parallel processing, assuming again stochastic independence across the items or channels, the overall completion time for both items has to be the last, or maximum finishing time for either item. Thus, the density that measures the last finishing time is *f*_max_(*t*) = *f_a_*(*t*)*F_b_*(*t*) + *f_b_*(*t*)*F_a_*(*t*). While *f*(*t*) represents the density function, *F*(*t*) represents the cumulative distribution function. The interpretation of this formula is that *a* is either the last to finish by time *t* (*b* is already done by then), or *b* finishes last at time *t* and *a* is already done by then. In this case, we can write the mean in terms of the survivor function: *E*[*T*] = ∫ *S*(*t*)*dt*, integrating *t* from 0 to infinity. The survivor function in the present situation is *S*(*t*) = 1 − *F_a_*(*t*)*F_b_*(*t*) and the mean can be calculated using the already given integral.

## Standard serial models

This type of model is what most people mean when they only say “serial unadorned.” Thus, it is the model advocated by Sternberg in many of his early papers (e.g., Sternberg, 1966). To reach it in the case that *n* = 2, let *f_a_*(*t*) = *f_b_*(*t*) = *f*(*t*). That is, the probability densities are the same across items or positions and even *n*. The latter indicates that *f*(*t*) defines the length of time taken on an item or channel no matter how the size of the set of operating items or channels. Furthermore, it is assumed in the standard serial model that each successive processing time is independent of all others. So, if *a* is second, say, its time does not depend on how long the preceding item (e.g., *b*) took to complete its processing.

Note, however, that we allow that different paths through the items might be followed from trial to trial. We also do not confine the stopping rule to a single variety. Now, Sternberg's preferred model assumed that exhaustive processing (all items were required to finish to stop) was used even in target-present trials. But we allow the standard model to follow other, sometimes more optimal, rules of cessation. Because all the *n* densities are now the same we can simply write the *n*th order convolution for exhaustive processing in symbolic form as *f*_max_(*t*) = f^*(*n*)^ (*t*). The exhaustive mean processing time is then *E*_max_[*T*_1_ + *T*_2_ + … + *T_n_*] = *nE*[*T*].

Next consider the situation where exactly one target is present among *n* − 1 distractors and the system is self-terminating (ST; only one item is required to stop the process). Again, it is assumed that the target is placed with probability 1/*n* in any of the *n* locations. Then it follows that *f_st_*(*t*) = 1/*n* ∑f^*(*i*)^. The mean processing time in this case is the well-known *E_st_*[*T*] = (*n* + 1)*E*[*T*]/2. This formula can be interpreted that on average, it takes the searcher approximately one-half of the set of items to find the target and cease processing. Finally, when processing stops as soon as the first item is finished, then we have the result *f*_min_(*t*) = *f*(*t*) and that *E*_min_[*T*] = *E*[*T*].

## Standard parallel models

The standard parallel model also assumes independence among the processing items, but this time in a simultaneous sense. Thus, the processing time on any individual channel is stochastically independent of that of any other channel. The standard parallel model further assumes unlimited capacity. The notion of capacity will be developed in detail below but suffice to mention for the moment that it means that, overall, the speed of each channel does not vary as the number of other channels in operation is varied. However, we do not assume that the various channel distributions are identical, unlike the standard serial model. Here, mean exhaustive processing time is just *E*[MAX(*T*_1_, *T*_2_, …, *T*_*n* − 1_, *T_n_*)] and the mean time in the event of single target self-termination and the target is in channel *i*, is simply *E*[*T_i_*]. That for the minimum time (i.e., race) is *E*[MIN(*T*_1_, *T*_2_, …, *T*_*n* − 1_, *T_n_*)].

## Selective influence

For decades, a popular way to attempt to test serial vs. parallel processing has been to vary the processing load (i.e., number of items, *n*), and then to plot the slopes of the mean response times as a function. If the slope of such a graph differs significantly from 0, then processing is declared to be serial. If it does not differ significantly from 0, parallel processing is inferred. This reasoning is fallacious on several grounds but the major infirmity is that such “tests” are primarily assessing capacity as workload changes, not architecture. Thus, what is commonly determined to be evidence for serial processing can be perfectly and mathematically mimicked by a limited capacity parallel model (Townsend, 1990; Townsend et al., 2011).

Sternberg's celebrated additive factors (Sternberg, 1969) method offered a technique which avoided the fragile capacity logic, which could affirm or deny serial processing. The method was based on the notion of “selective influence” of mean processing times, which stipulated that each experimental factor affect one and only one psychological subprocess at the level of means. The challenge there was that the method did not directly test other important architectures such as parallel systems. Also, there was a lack of mathematical proof for the association of “factors that are additive” even with serial processing if the successive times were not stochastically independent and again, no clear way to include other architectures.

Townsend and Schweickert (1989) proved that if selective influence acted at a stronger level, then many architectures, including parallel and serial ones, could be discriminated at the level of mean response times. Subsequent work, and that which we attempted to implement here, extended such theorems to the more powerful level of entire response time distributions (Townsend and Nozawa, 1995; Townsend and Wenger, 2004b).

We have discovered many tasks where stern tests of selective influence are passed. When they do not pass the tests it can itself often help to determine certain aspects of a processing system (see, e.g., Eidels et al., 2011). However, the strict use of the methodology to assess architecture cannot be applied. As we will learn below, the tests were not successfully passed, and this feature does play an important role in our discussion.

## Independence vs. dependence of channel or item processing times

We also must discuss *independence* vs. *dependence* of channels, stages, or subsystems (these terms can be used interchangeably although the term stages is sometimes restricted to serial systems and channels to parallel systems). In this introduction, we have been explicitly assuming stochastic independence of processing times, whether the architecture is serial or parallel.

In serial processing, if the successive items are dependent, then what happens on *a*, say, can affect the processing time for *b*. Although it is still true that the overall mean exhaustive time will be the sum of the two means, the second, say *b*, will depend on *a*'s processing time. Speeding up *a* could either speed up or slow down *b* because they are being processed simultaneously; ongoing inhibition or facilitation (or both) can take place during a single trial and while processing is ongoing. Townsend and Wenger (2004b) discuss this topic in detail.

It is interesting to note that the earlier prediction of independent parallel processing in self-terminating situations will no longer strictly hold. However, it will still be true even if processing is dependent that the predicted ST density will be the average or expected value of the density in the channel where the sought-for target is located, *E*[*T_a_*]. Only in the non-independent situation, this expectation has to be taken over all the potential influences from the surrounding channels.

## Stopping or decision rule: when does processing cease?

No predictions can be made about processing times until the model designer has a rule for when processing stops. In some high-accuracy situations, such as search tasks, it is usually possible to define a set of events, any one of which will allow the processor to stop without error. In search for a set of targets then, the detection of any one of them can serve as a signal to cease processing. A special case ensues when exactly one sought- for target is present. In any task where a subset of the display or memory items is sufficient to stop without error, and the system processor is capable of stopping (not all may be), the processor is said to be capacity of *self-termination*. Because many earlier (e.g., Sternberg, 1966) investigations studied exhaustive vs. single-target search, self-termination was often employed to refer to the latter, although it can also have generic meaning and convey, say, *first-termination* when the completion of any of the present items suffices to stop processing. The latter case is often called an OR design because completion of any of a set of presented items is sufficient to stop processing and ensure a correct response (e.g., Egeth, 1966; Townsend and Nozawa, 1995).

If all items or channels must be processed to ensure a correct response then exhaustive processing is entailed. For instance, on no-target (i.e., nothing present but distractors or noise) trials, every item must be examined to guarantee no targets are present. In an experiment where, say, all *n* items in the search set must be a certain kind of target, called an AND design, exhaustive processing is forced on the observer (e.g., Sternberg, 1966; Townsend and Nozawa, 1995). Nevertheless, as intimated earlier, some systems may by their very design have to process everything in the search set, so the question is of interest even when, in principle, self-termination is a possibility.

**Figure 2:**
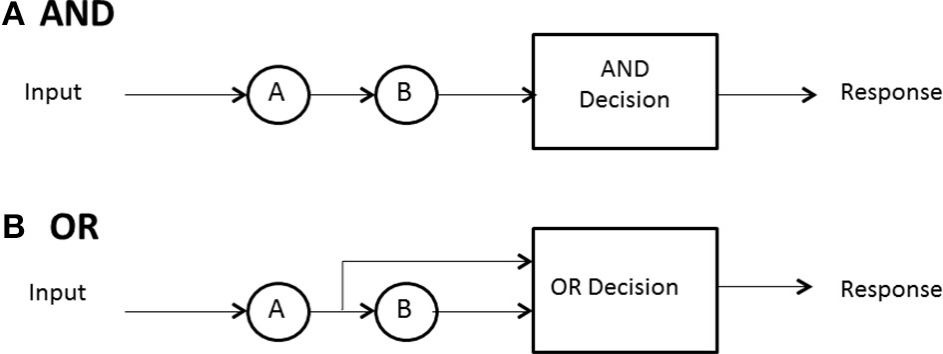
**Depiction of stopping rules in a serial system: (A) AND, (B) OR**.

**Figure 3:**
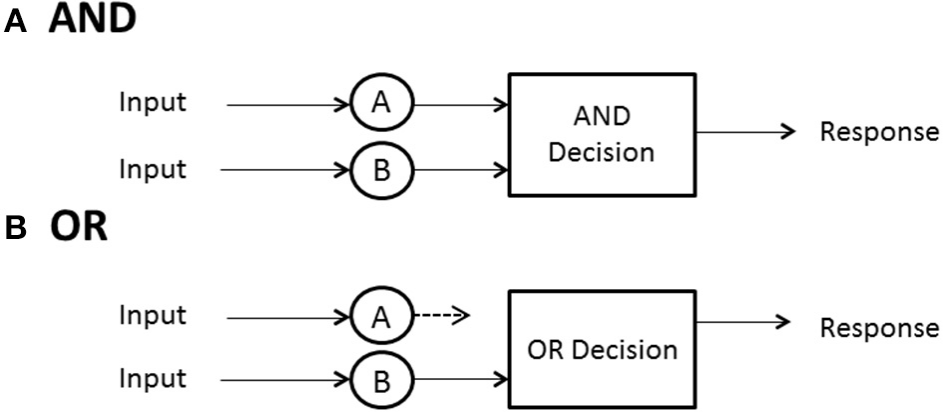
**Depiction of stopping rules in a parallel system: (A) AND, (B) OR**.

Hence, in summary, there are three cases of especial interest:(a) minimum time, OR, or first-self-termination, where there is one target among *n* − 1 other items and processing can cease when it is found; (b) single-target self-termination, where there is one target among *n* − 1 other items and processing can cease when it is found, and (c) exhaustive or AND processing, where all items or channels are processed. Figure 2 depicts AND (exhaustive) and OR (first-terminating) processing in a serial system, whereas Figure 3 does the same for a parallel system. Suppose again there are just two items or channels to process, *a* and *b*, and serial processing is being deployed. Assume that a is processed first. Then the minimum time processing density is simply *f*_min_(*t*) = *f_a_*(*t*), naturally just the density of *a* itself. Assume now there is a single target present in channel *a* and one distractor is in channel *b*, and self-terminating serial processing is in force. Then the predicted density is *f_st_*(*t*) = *pf_a_*(*t*) + (1 − *p*)*f_b_*(*t*)^*^f_a_(*t*). That is, if *a* happens to be checked first, which occurs with probability *p*, then the processing stops. On the other hand, if *b* is processed first and *a* distractor is found then *a* has to be processed also so the second term is the convolution of the *a* and *b* densities. In the event that both items must be processed, then the prediction is just that given earlier: *f*_max_(*t*) = *f_a_*(*t*)^*^f_b_(*t*).

When processing is independent parallel, the minimum time rule delivers a horse race to the finish, with the winning channel determining the processing time (Figure 3B). The density is just *f*_min_(*t*) = *f_a_*(*t*)*S_b_*(*t*) + *f_b_*(*t*)*S_a_*(*t*). This formula possesses the nice interpretation that *a* can finish at time *t* but *b* is not yet done (indicated by *b*'s survivor function), or the reverse can happen. If processing is single-target self-terminating with the target in channel *a*, parallel independence predicts that the density is the simple *f_st_*(*t*) = *f_a_*(*t*). Finally, if processing is exhaustive (maximum time) and independent, then processing is the same as shown before: *f*_max_(*t*) = *f_a_*(*t*)*F_b_*(*t*) + *f_b_*(*t*)*F_a_*(*t*) (Figure 3A).

The stopping rule in our experiments is always OR, that is, the observers were required to respond with the “yes” button if a signal tone appeared either in the left ear, the right ear, or both ears. Otherwise, they were instructed to respond with the “no” button.

## Capacity and workload capacity: various speeds on a speed continuum

*Capacity* generally refers to the relationships between the speeds of processing in response-time tasks. Workload capacity will refer to the effects on efficiency as the workload is increased. For greater mathematical detail and in-depth discussion, see Townsend and Ashby (1978), Townsend and Nozawa (1995), and Townsend and Wenger (2004b). Wenger and Townsend (2000) offer an explicit tutorial and instructions on how to carry out a capacity analysis.

**Figure 4:**
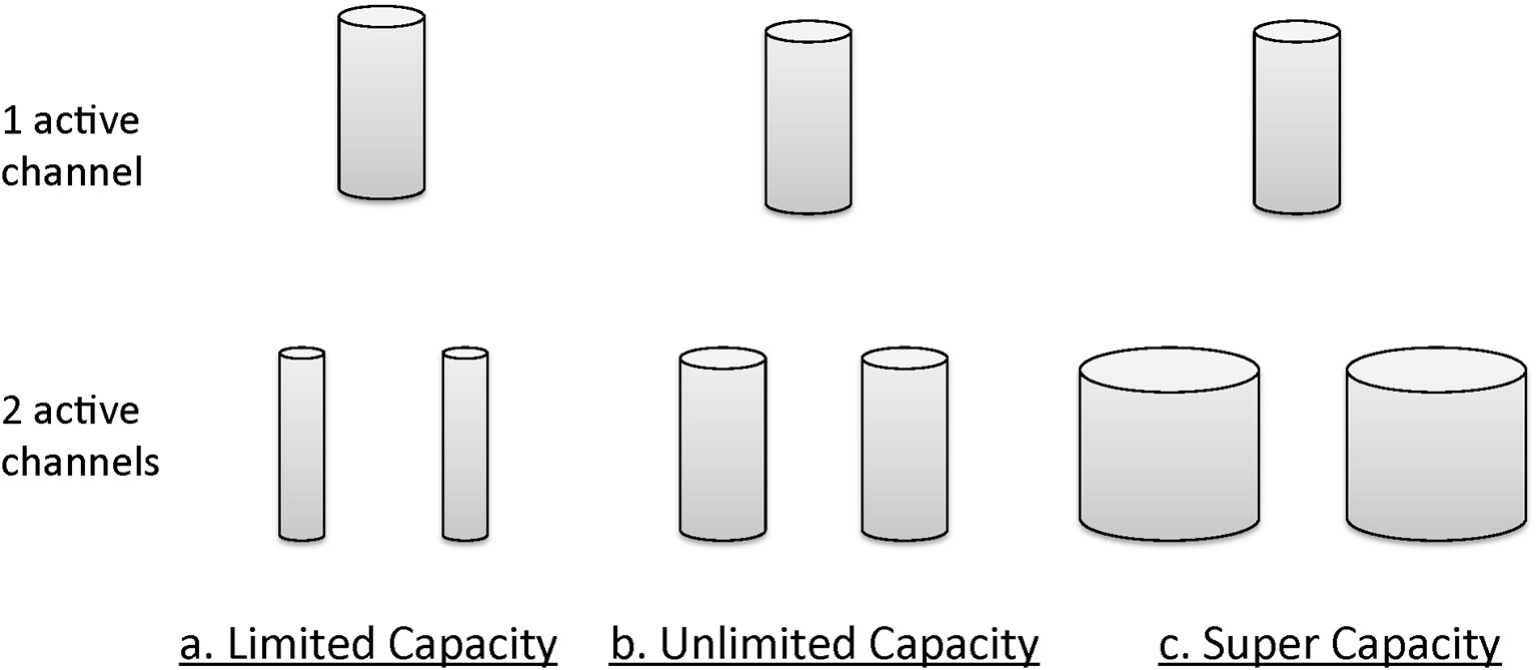
**Graphical intuition of a system's behavior under different capacity bounds: unlimited capacity, limited capacity, and supercapacity**.

Informally, the notion of *unlimited capacity* refers to the situation when the finishing time of a subsystem (item, channel, etc.) is identical to that of a standard parallel system (described in more detail later); that is, the finishing times of the distinct subsystems are parallel, and the average finishing times of each do not depend on how many others are engaged [e.g., in a search task the finishing time marginal density function for an item, channel etc., *f*(*t*) is invariant over the total number of items being searched]. *Limited capacity* refers to the situation when item or channel finishing times are less than what would be expected in a standard parallel system. *Super capacity* indicates that individual channels are processing at a rate even faster than standard parallel processing. Figure 4 illustrates the general intuitions accorded these concepts, again in an informal manner. The size of the cylinders provides a description of the amount of resources available.

**Figure 5:**
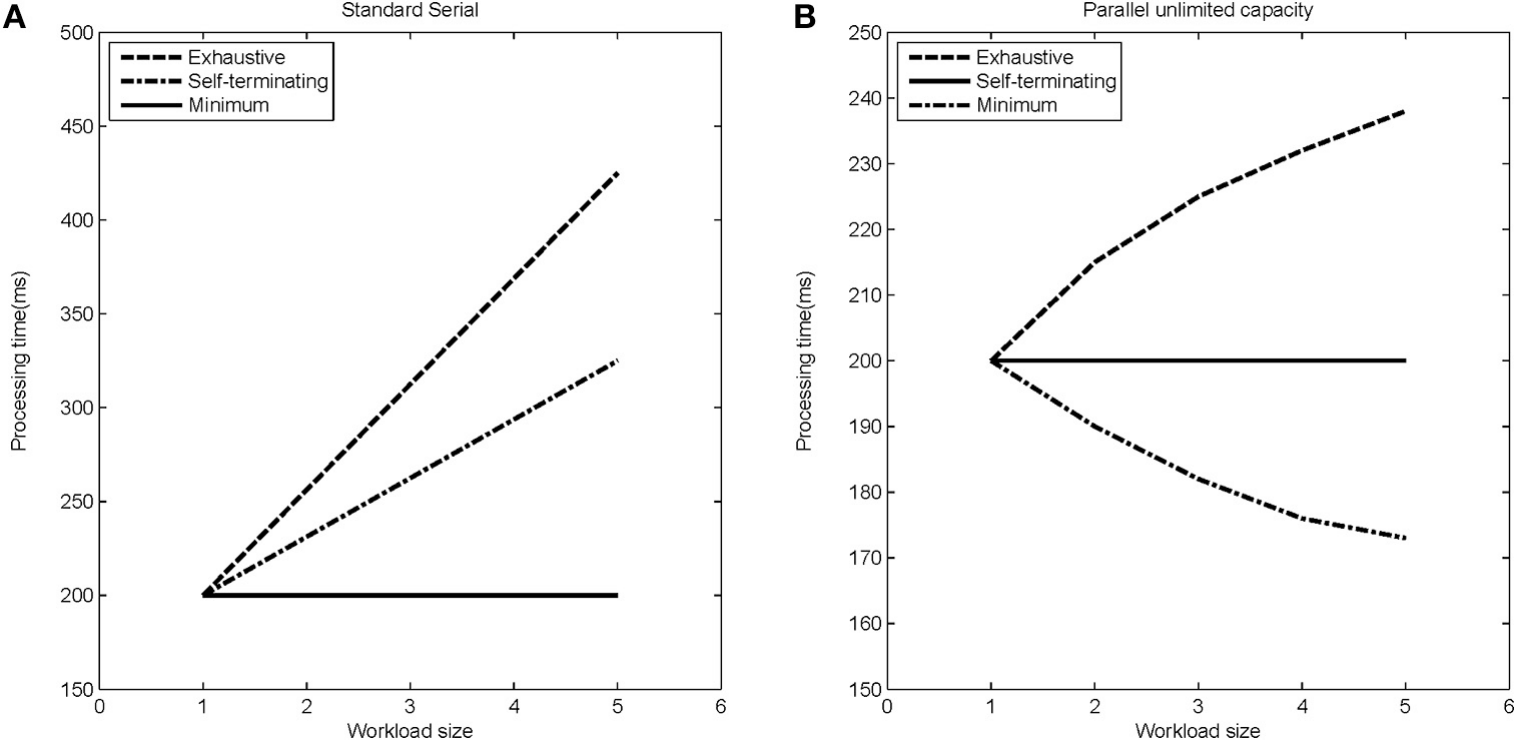
**Expected processing time as a function of load-set size for different stopping rules (exhaustive, self-terminating, and minimum) for (A) the standard serial modal, and (B) the parallel unlimited capacity processing model**.

The stopping rule obviously affects overall processing times (see Figure 5 for a depiction of how RTs change with increasing workload for the different models). Figure 5 indicates mean response times as a function of workload. *Workload* refers to the quantity of labor required in a task. Most often, workload is given by the number of items that must be operated on. For instance, workload could refer to the number of items in a visual display that must be compared with a target or memory item.

However, we assess capacity (i.e., efficiency of processing speed) in comparison with standard parallel processing with specification of a particular stopping rule. Thus, although the minimum time (first-terminating or OR processing) decreases as a function of the number of items undergoing processing (because all items are targets), the system is merely unlimited, not super, because the actual predictions are from a standard parallel model (i.e., unlimited capacity with independent channels). But observe that each of the serial predictions would be measured as limited capacity because for each stopping rule, they are slower than the predictions from standard parallel processing.

Although Figure 5 indicates speed of processing through the mean response times, there are various ways of measuring this speed. The mean (*E*[*T*]) is a rather coarse level of capacity measurement. A stronger gauge is found in the cumulative distribution function *F*(*t*), and the hazard function [*h*(*t*), to be discussed momentarily] is an even more powerful and fine grained measure. This kind of ordering is a special case of a hierarchy on the strengths of a vital set of statistics (Townsend and Ashby, 1978; Townsend, 1990).

The ordering establishes a hierarchy of power because, say, if *F_a_*(*t*) > *F_b_*(*t*) then the mean of *a* is less than the mean of *b*. However, the reverse implication does not hold (the means being ordered do not imply an order of the cumulative distribution functions). Similarly if *h_a_*(*t*) > *h_b_*(*t*) then *F_a_*(*t*) > *F_b_*(*t*), but not vice versa, and so on. Obviously, if the cumulative distribution functions are ordered then so are the survivor functions. That is, *F_a_*(*t*) > *F_b_*(*t*) implies *S_a_*(*t*) < *S_b_*(*t*).

There is a useful measure that is at the same strength level as *F* or *S*. This measure is defined as—ln *S*(*t*). Wenger and Townsend (2000) illustrate that this is actually the integral of the hazard function *h*(*t*′) from 0 to *t* (e.g., Wenger and Townsend, 2000; see also Neufeld et al., 2007). We thus write the integrated hazard function as *H*(*t*) = − log[*S*(*t*)]. Although *H*(*t*) is of the same level of strength as *S*(*t*), it has some very helpful properties not directly shared by *S*(*t*).

Now it has been demonstrated that when processing is of this form, the sum of the integrated hazard functions for each item presented alone is precisely the value, for all times *t*, of the integrated hazard function when both items are presented together (Townsend and Nozawa, 1995). That is, *H_a_*(*t*) + *H_b_*(*t*) = *H_ab_*(*t*). This intriguing fact suggests the formulation of a new capacity measure, which the Townsend and Nozawa called the *workload capacity coefficient* *C*(*t*) = *H_ab_*(*t*)/[*H_a_*(*t*) + *H_b_*(*t*)], that is, the ratio of the double item condition over the sum of the single item conditions. If this ratio is identical to 1 for all *t*, then the processing is considered *unlimited*, as it is identical to that of an unlimited capacity independent parallel model. If *C*(*t*) is less than 1 for some value of *t*, then we call processing *limited*. For instance, either serial processing of the ordinary kind or a fixed-capacity parallel model that spreads the capacity equally across *a* and *b* predicts *C*(*t*) = 1/2 for all times *t* > 0. If *C*(*t*) > 1 at any time (or range of times) *t*, then we call the system *super capacity* for those times. A tutorial on capacity and how to assess it in experimental data is offered in Wenger and Townsend (2000). In a recent extension of these notions, we have shown that if configural parallel processing is interpreted as positively interactive parallel channels (thus being dependent or positively correlated rather than independent), then configural processing can produce striking super capacity (Townsend and Wenger, 2004b).

Subsequently, a general theory of capacity was formulated that permitted the measurement of processing efficiency for all times during a trial (Townsend and Nozawa, 1995). Employing standard parallel processing as a cornerstone, the theory defined unlimited capacity as efficiency identical to that of standard parallel processing in which case the measure is *C*(*t*) = 1. It defined limited capacity as efficiency slower than standard parallel processing. For instance, standard serial processing produces a measure of capacity of *C*(*t*) = 1/2. And finally, the theory defined super capacity as processing with greater efficiency than standard parallel models could produce, that is, *C*(*t*) > 1.

In sum, our measuring instrument is that of the set of predictions by unlimited-capacity independent parallel processing (UCIP). As mentioned above, *unlimited capacity* means here that each parallel channel processes its input (item, etc.) just as fast when there are other surrounding channels working (i.e., with greater n) as when it is the only channel being forced to process information. The purpose of this paper is to apply these techniques, with a focus on comparing binaural detection capacity measures in diotic and dichotic contexts.

## Methods

### Stimuli

Stimuli were 440-Hz pure tones added to wide bands of noise. The target signal was a 250-ms pure tone with 25-ms cosine-squared onset and offset ramps. For each trial, the signal was generated with a random phase, selected according to a uniform distribution. The 500-ms noise was generated using a Gaussian distribution in the time domain at a sampling rate of 48828 Hz. A new random sample of noise was generated for each trial. The noise was always presented at a sound pressure level of 57 dB SPL and also had 25-ms rise/fall times. The target tone was presented at signal-to-noise ratios (SNR) of either +6 (the High SNR) and −6 dB (the Low SNR). These SNRs would be expected to yield accuracy measures near 100% for all detection conditions. Accuracy was indeed very high for all conditions and subjects: ranging from 97.5 to 99% percent correct.

### Procedures

On each trial, there were four possible events: a tone + noise presented to both ears (binaural trials), a tone + noise presented to the left ear, a tone + noise presented to the right ear, or noise alone. These four events were equally probable and are described below and are also illustrated in Table 1.

**Table 1:** **Illustration of stimulus conditions**.

	**Left ear**	**Right ear**	
Yes trials: dual targets (binaural)	S + N (High)	S + N (High)	HH
	S + N (High)	S + N (Low)	HL
	S + N (Low)	S + N (High)	LH
	S + N (Low)	S + N (Low)	LL
Yes trials: single targets (monaural)	S + N (High)		
	S + N (High)		
	S + N (Low)		
	S + N (Low)		
		S + N (High)	
		S + N (High)	
		S + N (Low)	
		S + N (Low)	
No trials (noise alone)	N	N	
	N	N	
	N		
		N	

*Each row represents an occurrence with frequency of 1/16th. S + N refers to signal + noise, N refers to noise, and a blank space indicates no stimulus presented. H and L refer to High and Low signal-to-noise ratios, respectively. Seventy-five percent of the trials are “Yes” (signal-present trials) whereas 25% of the trials are “No” (signal-absent trials)*.

In the tone + noise trials (“Yes” trials), the SNR was manipulated such that the low and the high SNRs were presented equally often. The binaural trials (referred to as dual-target trials) yield four possible events (see Table 1, top four rows): Left ear-High + Right ear-High (denoted HH throughout), Left ear-High + Right ear-Low (HL), and Left ear-Low + Right ear-High (LH), Left ear-Low + Right ear-Low (LL). The monaural trials (referred to as single-target trials) yielded two SNRs (High and Low) for each ear. These are depicted in the middle eight rows of Table 1.

Of the noise (or “No”) trials, 1/2 of the trials presented the noise in both ears, 1/4 of trials had noise in the left ear, and 1/4 of trials had noise in the right ear^1^. Trials were presented in random order throughout the experiment in blocks of 128 trials. Ten blocks were collected for each context, yielding a total of 80 trials in each dual-target condition (HH, LL, LH, HL) and 160 trials in each single-target condition (Left-High, Left-Low, Right-High, Right-Low).

Trials were run in two separate contexts, defined by the characteristics of the dual-target trials: *N*_0_*S*_0_ and *N*_0_*S*_π_. In the *N*_0_*S*_0_ context (diotic), identical noises and signals were presented to the two ears. In the *N*_0_*S*_π_ context (dichotic), the noises were identical across the ears but the signal was phase shifted by π radians to one of the ears. Note that the single-target stimuli were the same regardless of whether they were presented in the *N*_0_*S*_0_ or *N*_0_*S*_π_ context. In this way, a single block in either context consisted of 50% single-target trials (½ to left ear and ½ to right ear), 25% dual-target trials, and 25% noise-alone trials.

Observers participated in experimental sessions lasting 1 h. A single session consisted of 6–8 blocks of 128 trials. Each trial began with a visual warning of “listen” appearing on a computer monitor for 500 ms. A silent period of 500 ms followed removal of the warning, when the noise stimulus began. When the 250-ms target tone was present, it occurred at a random interval from 50 to 250 ms after the onset of the 500-ms noise.

Stimuli were presented to the observers at a 24414 kHz sampling rate using a 24-bit Tucker Davis Technologies (TDT) RP2.1 real-time processor. Target and masker were summed digitally prior to being played though a single channel of the RP2.1 (for the monaural stimuli) or both channels of the RP2.1 (for the binaural stimuli). Each channel was calibrated via a PA5 programmable attenuator, passed through an HB6 headphone buffer, and presented to observers through a Sennheiser HD280 Pro headphone set. Reaction times were measured using a button box interfaced to the computer through the TDT hardware.

### Observers

Four listeners, ranging in age from 20 to 43 participated in the experiment. All subjects had hearing thresholds of 15 dB HL or better in both ears at all audiometric frequencies. Obs. 4 is the first author. Obs 1–3 competed trials in the *N*_0_*S*_0_ context first whereas Obs. 4 completed trials in the *N*_0_*S*_π_ context first. Subjects provided written informed consent prior to participation and Obs. 1–3 were paid per session. Testing procedures were overseen by Indiana University's Institutional Review Board.

Observers were instructed to respond as quickly to the signal tone as possible while attempting to provide correct responses. Using an “OR” design, observers were required to respond with the “yes” button if a tone was present. Otherwise, they were instructed to respond with the “no” button. The RT was measured from the onset of the tone stimulus within the noise. Percent correct was recorded in order to ensure that subjects achieved high levels of performance for both SNRs.

## Results

### Mean reaction times

Table 2 shows mean RTs in milliseconds for single targets for the two contexts (*N*_0_*S*_0_ and *N*_0_*S*_π_). Reaction times below 100 ms or greater than 3 standard deviations from the mean were excluded from the data set. A repeated-measures ANOVA revealed a significant effect of SNR [*F*_(1, 3)_ = 586.6, *p* < 0.0001] in which faster RTs were associated with the higher SNR (254 vs. 209 ms). No other significant main effects or interactions were revealed by the ANOVA, although the main effect of context approached significance [*F*_(1, 3)_ = 10.0; *p* = 0.051]. The slightly faster RTs in *N*_0_*S*_π_ (293 vs. 270 ms) may be due to three of the observers completing *N*_0_*S*_π_ after *N*_0_*S*_0_ and consequently could be attributable to practice effects. However, even Obs. 4 was faster in *N*_0_*S*_π_ and she completed these conditions first. Recall that for these contexts, the same stimuli were used for the single-target conditions, and so no difference in context was expected.

**Table 2:** **Mean reaction times in ms for the single-target conditions for each subject in the two contexts**.

	***N*_0_*S*_0_**				***N*_0_*S*_π_**			
	**Left ear**		**Right ear**		**Left ear**		**Right ear**	
	**Low**	**High**	**Low**	**High**	**Low**	**High**	**Low**	**High**
Obs 1	289	232	291	228	278	226	276	225
Obs 2	310	272	318	266	305	252	304	254
Obs 3	316	259	313	254	281	228	290	230
Obs 4	371	295	350	317	332	262	320	265
Average	321 (17)	265 (13)	318 (12)	266 (19)	299 (13)	242 (9)	297 (9)	243 (10)

*RTs for both ears and both SNRs are shown. Standard errors of the mean are indicated for the averages*.

These results are consistent with previous studies demonstrating a robust negative relationship between the RT and the intensity of the stimulus being detected in quiet (e.g., Chocholle, 1944; Kohfeld, 1971; Grice et al., 1974; Santee and Kohfeld, 1977; Schlittenlacher et al., 2014) as well as the signal-to-noise ratio (and signal levels) for a signal detected in noise (e.g., Green and Luce, 1971; Kemp, 1984). Accuracy was very high, with the miss rate averaging 0.5% for the high SNR and 2.6% for the low, also implicating a small difference in accuracy for the two SNRs. Consequently, we, like others, have observed strong selective influence effects for single-target stimuli.

Table 3 shows the mean RTs in milliseconds for the dual target conditions for *N*_0_*S*_0_ and *N*_0_*S*_π_ contexts. A repeated-measures ANOVA revealed a significant effect of SNR [*F*_(3, 9)_ = 95.8, *p* < 0.0001] and an interaction between context and SNR [*F*_(3, 9)_ = 18.7; *p* < 0.001]. *Post-hoc t*-tests with a Bonferroni correction indicated that RTs in LL were slower than all other conditions, but only for *N*_0_*S*_0_.

**Table 3:** **Mean reaction times in ms for the dual-target conditions**.

	***N*_0_*S*_0_**				***N*_0_*S*_π_**			
	**HH**	**LL**	**LH**	**HL**	**HH**	**LL**	**LH**	**HL**
Obs 1	225	266	229	228	222	244	218	225
Obs 2	255	306	262	259	252	273	253	263
Obs 3	247	312	257	257	213	243	223	226
Obs 4	299	344	300	306	260	280	273	266
Average	257 (15)	307 (16)	262 (15)	263 (16)	234 (12)	260 (10)	242 (13)	245 (11)

*Standard errors of the mean are indicated in parentheses for the averages*.

For the *N*_0_*S*_0_ context, a general failure of selective influence is evident, as only LL was associated with RTs slower than the other conditions. Recall that for accuracy data, *N*_0_*S*_0_ detection thresholds are similar to monaural (*N_m_S_m_*) detection thresholds. Thus, these RT results essentially mirror the threshold data: HH, LH, and HL RTs are effectively determined by the faster of the two detections. For LH and HL, this is the stimulus with the higher SNR. Note, however, there is a slight (albeit not statistically significant) trend for the HH trials to have faster RTs than the HL and LH trials. On average, the HH trials are about 5 ms faster than the HL and LH trials. If we consider that HL and LH trials are similar to monaural presentation, we see that this result is similar to the size of the effect observed for monaural vs. binaural stimulation for pure tones (e.g., Chocholle, 1944; Simon, 1967; Schröter et al., 2007 Exp. 1; Schlittenlacher et al., 2014). Although the effect size, as measured by Cohen's d, is less than 0.2 we believe that with more samples we would see a consistent advantage of two ears over one in mean RT.

Further, there is some evidence that RTs are faster in for the dual targets than for the single targets. In the *N*_0_*S*_0_ context, RTs for the high SNR were 257 ms for the HH dual targets and 265 ms for the High single targets. For the low SNR, RTs were 307 ms for the LL dual targets and 320 ms for the Low single targets. These results again imply a small but consistent binaural advantage for detecting tones embedded in noise. Miss rates also followed this trend, averaging 0.5% for dual targets and 1.6% for single targets.

In the *N*_0_*S*_π_ context, we see failure of selective influence, with no statistically significant difference between any of the dual-target conditions. These results do not simply suggest that the RT is primarily driven by the stimulus yielding the faster RT because RTs in LL are similar to those in HH. Here, mean RTs for the LL conditions are significantly faster for the dual target than the single-target conditions. RTs for LL were 260 ms but were 298 ms for the low-SNR single targets. The implications of these results will be discussed subsequently, as we address the RT distributions and in the section describing capacity. Miss rates were 0% for all subjects and conditions within *N*_0_*S*_π_.

### Survivor functions

**Figure 6:**
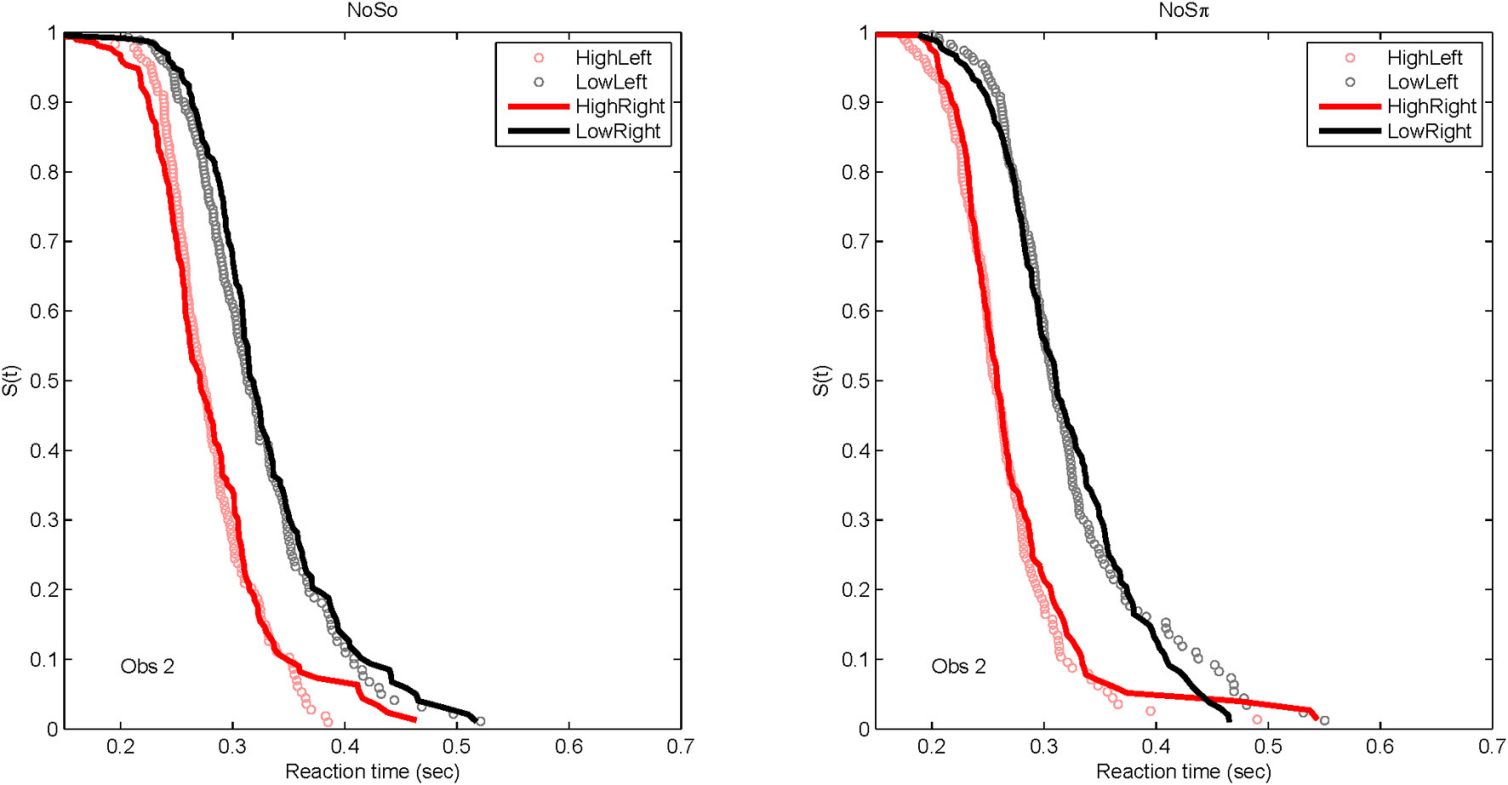
**Derived survivor functions for the single-target conditions at the two SNRs for the left and right ears in the two contexts: *N*_0_*S*_0_ (left panels) and *N*_0_*S*_π_ (right panels) for a single representative subject**.

Although of primary interest to this paper are the RT data for the dual target conditions, it is worth presenting the RT distributions for the single-target data, to familiarize the reader to the data format and to present the robust reaction-time distributional data. Figure 6 plots derived survivor functions for the high and low SNRs presented to the left and right ears in the two contexts: *N*_0_*S*_0_ (left panels) and *N*_0_*S*_π_ (right panels). Recall that the survivor function, *S*(*t*) is simply 1 − *F*(*t*), where *F*(*t*) represents the cumulative distribution function of RTs. Data from a representative single subject (Obs. 2) are presented because of overwhelming similarity in the pattern of results across the subjects.

Because a powerful ordering of faster RTs associated with the high SNR ratio, the same symbols are used to display data from the left ear (unfilled circles) and data from the right ear (solid lines). All subjects demonstrated significantly faster RTs for the high SNRs vs. the low SNR. For all statistical tests, non-parametric Kolmogorov-Smirnov (KS) tests of survivor function orderings at the *p* < 0.0001 level were taken to establish statistical significance. The lower-than-typically used *p-*value is used due to the presence of multiple comparisons. The only parameter associated with survivor function ordering was SNR. Table 4 presents the *p*-values to illustrate the pattern of results across subjects. There also was no difference in RTs measured for the single targets dependent on context. That is, the RT distributions for single targets were not statistically different whether RTs were measured in the *N*_0_*S*_0_ or the *N*_0_*S*_π_ context.

**Table 4:** ***p*-values for Kolmogorov–Smirnov (KS) test for single targets**.

		**Left**	**Right**	**High**	**Low**
		**Low vs. High**	**Low vs. High**	**Left vs. Right**	**Left vs. Right**
*N*_0_*S*_0_	Obs 1	<0.0001^**^	<0.0001^**^	0.47	0.65
	Obs 2	<0.0001^**^	<0.0001^**^	0.12	0.20
	Obs 3	<0.0001^**^	<0.0001^**^	0.47	0.65
	Obs 4	<0.0001^**^	<0.0001^**^	0.02	0.32
*N*_0_*S*_π_	Obs 1	<0.0001^**^	<0.0001^**^	0.56	0.91
	Obs 2	<0.0001^**^	<0.0001^**^	0.91	0.56
	Obs 3	<0.0001^**^	<0.0001^**^	0.65	0.25
	Obs 4	<0.0001^**^	<0.0001^**^	0.47	0.75

** *Indicates statistical significance at the p < 0.0001 level*.

The data present a compelling case that selective influence is present for tone-in-noise detection and that increases in SNR facilitate a faster RT. Further, the context in which the RTs were measured (in the presence of *N*_0_*S*_0_ or *N*_0_*S*_π_ stimuli) has little effect on the distribution of RTs. We also see no evidence that the right ear is faster than the left ear for tone-in-noise detection, at least in a task where listeners must divide their attention across ears (see also Schlittenlacher et al., 2014).

**Figure 7:**
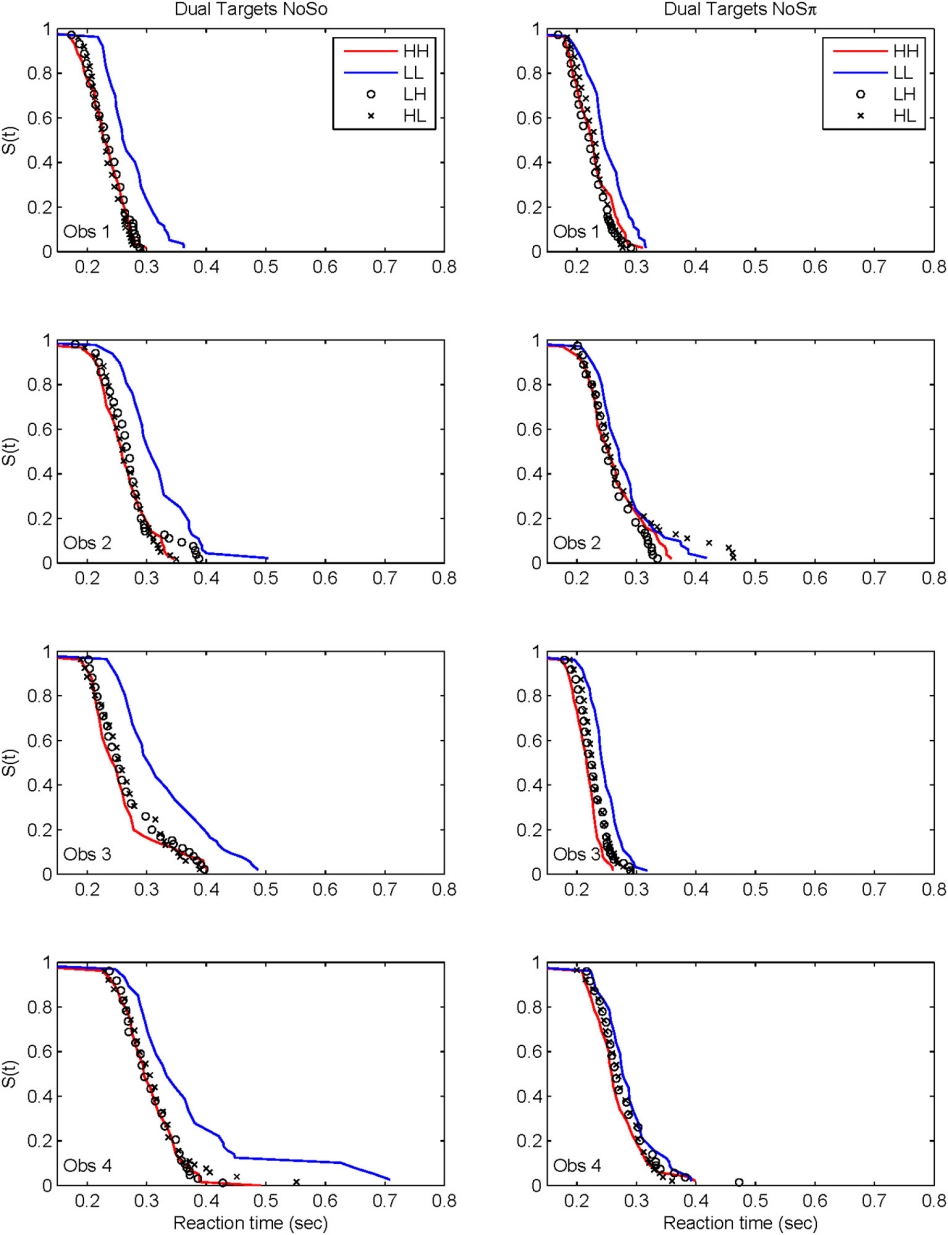
**Derived survivor functions for the dual-target conditions in the two contexts: *N*_0_*S*_0_ (left panels) and *N*_0_*S*_π_ (right panels)**.

Figure 7 plots the derived survivor functions for the dual target data in the *N*_0_*S*_0_ contexts (left panels) and the *N*_0_*S*_π_ contexts (right panels). For all observers, a failure of selective influence is obvious, with HH, HL, LH being not statistically different from each other. This overlap is present for both the *N*_0_*S*_0_ contexts and the *N*_0_*S*_π_ contexts.

The *N*_0_*S*_π_ contexts reveal a slightly different pattern although the failure of selective influence is still obvious. The only consistent pattern across all subjects is LL < HH. Obs. 1, 3, and 4 show a pattern similar to *N*_0_*S*_0_ with LL < LH = LH. Obs. 4 also demonstrates HH < LH.

Although the *N*_0_*S*_π_ context indicates survivor function orderings that are a little more diverse across observers than the *N*_0_*S*_0_ context, the glaring failure in both immediately renders untenable any analysis of architecture. We shall discuss potential reasons for this failure in the General Discussion. In any case, the statistical function, *C*(*t*) = workload capacity, turns out to be highly informative all by itself.

### Capacity

**Figure 8:**
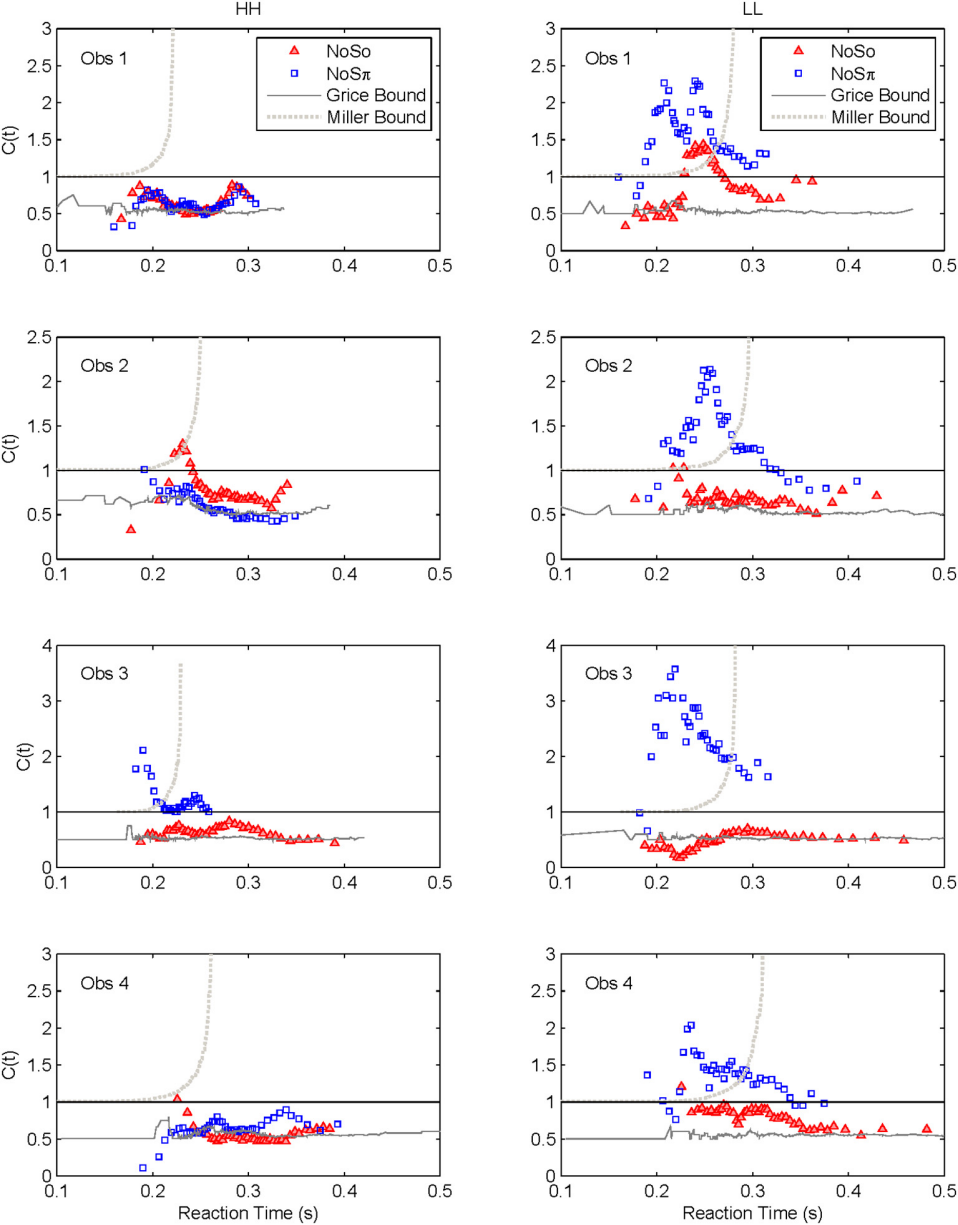
**Capacity functions for the two contexts are shown for HH and LL conditions**.

**Figure 9:**
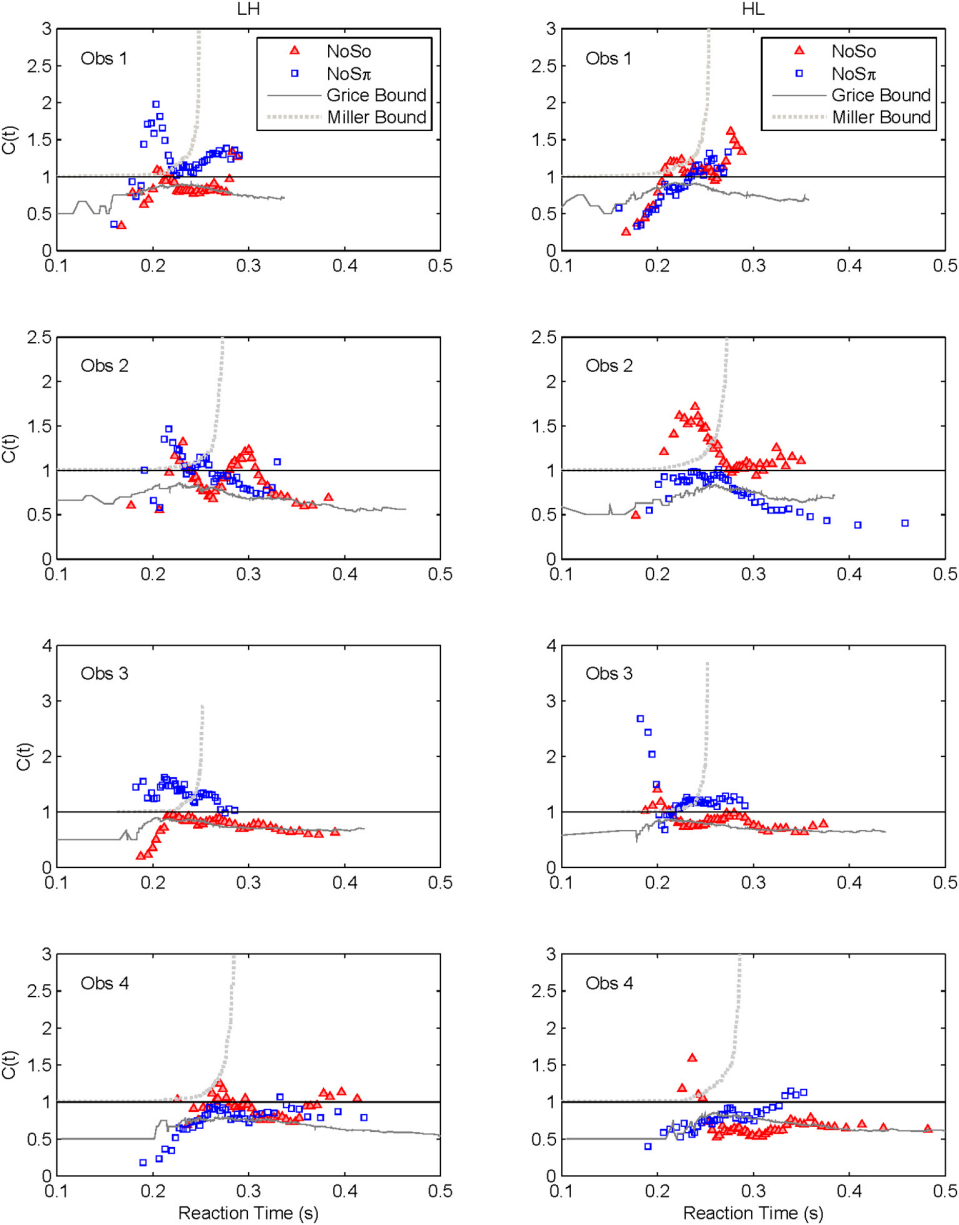
**Capacity functions for the two contexts are shown for LH and HL conditions**.

Capacity functions for the two contexts are plotted in Figures 8, 9 for the four subjects and summarized in Table 5 using Houpt and Townsend's (2012) statistical analysis. Because the HH and LL conditions showed the starkest contrast from one another, those are shown in Figure 8. Capacity functions for the LH and HL conditions are then shown in Figure 9.

**Table 5:** **Statistical inferences for the capacity functions**.

	***N*_0_*S*_0_**				***N*_0_*S*_π_**			
	**HH**	**LL**	**LH**	**HL**	**HH**	**LL**	**LH**	**HL**
Obs. 1	Limited^**^				Limited^**^	Super^*^	Super	
Obs. 2	Limited^**^	Limited^**^		Super	Limited^**^	Super		Limited^**^
Obs. 3	Limited^**^	Limited^**^	Limited^**^	Limited^*^		Super^**^	Super	
Obs. 4	Limited^**^		Limited^**^	Limited^**^	Limited^**^	Super		

*Cases where the null hypothesis (the Unlimited Capacity Independent Parallel model) can be rejected using the Houpt and Townsend (2012) statistical tests are displayed in the table with asterisks. Other cases trending toward limited (C consistently less than 0.8) and trending toward super capacity (C consistently greater than 1.25) are also indicated but without the asterisks indicating statistical significance*.

* P < 0.01;

** *P < 0.001*.

Miller (1982) suggested an inequality, or upper bound on RTs for channels involved in a race within a redundant-target paradigm. Consider the OR paradigm, where any target item can lead to a correct response, and suppose that the stimulus presentation initiates a race in a parallel system. The logic behind the Miller inequality states that if the marginal finishing time distributions from the single target conditions stay unchanged in the redundant target condition (implying unlimited capacity), then the cumulative distribution function for the double-targets display cannot exceed the sum of the single-target cumulative distribution functions (see, e.g., Townsend and Wenger, 2004b).

In our current language, violation of the Miller bound (i.e., the inequality), would imply super capacity. Next, it is possible, using a formula introduced by Townsend and Eidels (2011), to allow the investigator to plot this upper bound (referred to as the “Miller bound”) in the capacity space of Figures 8, 9. This tactic permits us to provide a direct comparison between the race model prediction and our data all within the same graph.

Grice and colleagues proposed a lower bound on performance parallel systems (e.g., Grice et al., 1984) that plays a role analogous to the Miller bound, but for limited as opposed to super capacity. If the Grice inequality is violated, the system is limited capacity in a very strong sense (Townsend and Wenger, 2004b). In this case, performance on double-target trials is slower than on those single-target trials that contain the faster of the two targets. When performance on the two channels is equal, the Grice bound indicates efficiency at the level of *fixed capacity* in a parallel system. A fixed capacity system can be viewed as sharing a fixed amount of capacity between the two channels. Alternatively, a serial system can make exactly this prediction as well (Townsend and Wenger, 2004b). This Grice boundary is also plotted on Figures 8, 9.

Across both figures and panels, the results for *N*_0_*S*_0_ consistently demonstrate *C*(*t*) ≤ 1, and the Miller bound is rarely exceeded by any of the capacity functions in the *N*_0_*S*_0_ context. Further, capacity tends to be at or slightly better than the Grice bound. Table 5 also shows that for all *N*_0_*S*_0_ conditions, at least two observers show statistically significant limited capacity [i.e., *C*(*t*) is significantly below 1].

Conversely, *N*_0_*S*_π_ data illustrate *C*(*t*) ≥ 1 over most of the RT range, and many *C*(*t*) values exceed the Miller bound in the *N*_0_*S*_π_ context, for LL particularly, implicating super capacity at the level where *C*(*t*) is much larger than 1 for longer RTs (see Townsend and Wenger, 2004b). Only the HH condition demonstrates significant limited capacity consistent across subjects. In the LL conditions, all observers reveal higher workload capacity in the *N*_0_*S*_π_ condition than in the *N*_0_*S*_0_ condition and in fact, the *N*_0_*S*_π_ *C*(*t*)s are higher than any of the other *C*(*t*) data, disclosing super capacity in all cases. Super capacity is statistically significant for two subjects in the *N*_0_*S*_π_ conditions, but only for LL. We believe that the other two subjects (Obs. 2 and 4) demonstrate evidence leaning toward super capacity but that there are limitations due to the sample size. Here, approximately 80 trials were used in each double-target condition. An examination of Houpt and Townsend (2012)'s Figure 4 suggests that more trials may be needed to establish significance of capacity in the 2.0 range. At a minimum, visual inspection indicates a difference among capacity functions, with the LL functions being above 1 and two of the four subjects demonstrating statistically significant super capacity. These two subjects also had data exceeding the Miller bound for many RTs, implicating capacity values that exceed race-model predictions.

### The high-low and low-high conditions

We lump these two conditions together since their results are very similar, though not identical. Interestingly, several observers appear to exhibit some super capacity, especially in the *N*_0_*S*_π_ conditions. By and large, *N*_0_*S*_0_ *C*(*t*) functions fall in the moderately limited capacity range, although there are spots of extremely limited capacity, for instance, Obs. 1 in both conditions, Obs. 2 in HL for slower times, Obs. 3 and 4 in LH early on. Although these tend to be concentrated in *N*_0_*S*_0_ trials, some pop up in *N*_0_*S*_π_ data.

In sum, all our statistics confirm that performance in *N*_0_*S*_0_ is very poor in comparison to *N*_0_*S*_π_ and in fact is close to being as poor as ordinary serial processing would predict. *N*_0_*S*_π_, on the other hand, regularly produces super capacity with the strongest and most consistent power in the slowest combination of factors (i.e., LL).

## General discussion

Up to this point, only para-threshold, accuracy experiments have investigated the binaural release from masking using pure tone detection in anti-phase. In fact, as mentioned in the introduction, only a handful of experiments have even employed RT at all when comparing binaural to monaural performance. This study presents analogs to the traditional accuracy statistics RTs for binaural auditory perception and in particular, for the first time, to the masking release effect.

Traditionally, detection thresholds have been the psychophysical tool in this domain. More generally, the psychometric functions can be analyzed from the point of view of probability summation (with appropriate corrections for guessing). We suggest that the appropriate RT analog to probability summation is what is termed the standard parallel model. This model, like probability summation, assumes that each channel acts the same way with one signal as it does with other channels operating at the same time (this is the unlimited capacity assumption). The standard parallel model also stipulates stochastic independence among the channels. It makes the probability summation prediction when only accuracy is measured.

First, although our experiment factor, SNR, was effectual in properly ordering the single-target survivor functions, it failed massively on the double signal trials: While HL, LH, and HH were all stochastically faster than LL (their survivor functions were all greater than that for LL for all times t), the former were very similar for almost all of our data and observers. The consequence is that we may not legitimately attempt to uncover the operational architecture in this experiment. However, the way in which selective influence fails plays a strategic role in our conclusions about the binaural processing system. From here on out, we will concentrate on other issues and especially that of capacity.

Next, recall that the single signal RT data are employed to assess the binaural data relative to predictions from the standard parallel model. If *C*(*t*) = 1, then performance is identical to that from the parallel model for that particular *t*, or range of *t*. If *C*(*t*) < 1, then limited capacity is concluded. If *C*(*t*) > 1, performance is super capacity relative to the standard parallel expectations. A somewhat more demanding upper bound is found in the Miller inequality, which nevertheless must be violated if *C*(*t*) exceeds 1 for intervals of the faster time responses (see Townsend and Nozawa, 1995). If the lower bound put forth by Grice and colleagues is violated, then capacity is very limited indeed. When performance on the two ears is equal, then the Grice bound is equivalent to *C*(*t*) = ½. On the other hand, if *C*(*t*) is even a little larger than the Grice bound, performance is said to show a redundancy gain. Finally, limited capacity could be associated with inadequate processing (e.g., attentional) resources or interfering channel crosstalk in a parallel system. If capacity is severely limited [e.g., *C*(*t*) < ½] it might be caused by serial processing, extreme resource deficits or even across-channel inhibition.

### Interpretation of *N*_0_*S*_0_ results

The results indicated that capacity typically was unlimited to severely limited in *N*_0_*S*_0_ conditions. At least two observers demonstrated limited capacity for each of the SNR combinations with all observers demonstrating limited capacity for HH. Potentially, there is more evidence for limited capacity in the HH conditions relative to the other conditions, though there is considerable variability across individuals in the value of the *C*(*t*) function and with respect to the *C*(*t*) functions proximity to the Grice bound.

The only other research of which we are aware, that has applied concepts from the redundant signals RT approach to binaural perception is a seminal study by Schröter et al. (2007) and extended in Schröter et al. (2009) and Fiedler et al. (2011). Schröter et al. (2007) employed the Miller (1982) inequality to assess binaural vs. monaural performance but did not assess performance in terms of the standard parallel model or the Grice bound for extreme limited capacity. They also did not address the antiphasic release-from-masking effect. Thus, we will be able to compare our *N*_0_*S*_0_ results to some extent with their results but not our *N*_0_*S*_π_ findings.

First, although we observed considerable individual differences in the capacity functions across listeners, a common trend was that in the *N*_0_*S*_0_ conditions, *C*(*t*) never exceeded 1. In many cases, *C*(*t*) was found to be significantly less than 1. In no instances was the Miller bound surmounted. Many of the capacity functions are also very similar to the Grice bound and display capacity values around 0.5, or fixed capacity. These results suggest that *a negligible gain* is provided by the addition of a second ear. These capacity values are also consistent with previous work demonstrating a very small two-ear advantage in mean RT (Chocholle, 1944; Simon, 1967; Schlittenlacher et al., 2014). Schröter et al. (2007) also demonstrated an almost complete lack of redundancy gain when identical pure tones were presented to each ear. Our data take their results a step further and report capacity values at two different SNRs. Although this conclusion is a tempered one, it is possible that the easiest to detect stimuli (High SNRs) yield the greatest degree of limited capacity.

This interpretation is closely associated with the trends present in the *N*_0_*S*_0_ survivor functions: the dual-target HH, HL, and LH survivor functions were virtually identical, even though SNR ordered the RT distributions for the single-target conditions (faster RTs for the High conditions). Thus, capacity should be more limited for HH than for HL or LH. It seems likely that the auditory system cannot take advantage of the addition of redundant well-defined signals, and may respond most prevalently to the “loud” or better-defined stimulus in these cases. These results very closely mirror those found in the threshold data, where only a negligible advantage is provided when a second ear is added to tone-in-noise detection tasks.

At this point, we cannot establish whether the lack of redundancy gain is due to interactions between the ears or true limitations in resource capacity. The presence of interactions in the auditory binaural pathway at every level in the auditory pathway central to the cochlear nucleus, indicates that interactions between the ears are prevalent. These interactions include both excitatory and inhibitory pathways, and are responsible for a complex and highly successful noise-reduction system. It appears, from detection and now RT data, the noise-cancelation properties of the auditory system are not activated when the ear receive the same signal and noise.

### Interpretation of *N*_0_*S*_π_ results

The *N*_0_*S*_π_ data reflect a different pattern of results than observed in the *N*_0_*S*_0_ contexts. First, two of the four subjects showed statistically significant levels of super capacity, with all four subjects leaning in that direction. This result occurred only in the LL conditions, but capacity was still higher for *N*_0_*S*_π_ than *N*_0_*S*_0_ for LH and HL. The intermediate conditions (HL and LH) tended toward unlimited capacity. Although one interpretation might be to treat the unlimited capacity functions as support for an independent, parallel model, it seems unlikely that such a model can also account for the limited capacity data observed for HH and the super capacity data observed for LL. Further it is commonly accepted that the BMLD occurs due to interactions between the two ears, and cross-correlation and equalization-cancelation are commonly employed tools implemented into binaural models (e.g., Bernstein et al., 1999; Davidson et al., 2009).

Our data reveal something that would not have been observed by using data obtained at threshold levels: an SNR-dependent effect at high accuracies. Traditionally, psychometric functions for *N*_0_*S*_0_ and *N*_0_*S*_π_ are treated as being parallel (e.g., Egan et al., 1969; Yasin and Henning, 2012). That is, the size of the BMLD does not depend on the accuracy. The implication, then, is that because the psychometric functions have the same shape and only shifted means, there are no SNR-dependent processes at play, although a few studies have demonstrated that the MLD decreases at very high signal sensation levels (e.g., Townsend and Goldstein, 1972; Verhey and Heise, 2012). By testing the binaural system at SNRs occurring well into the tip of the psychometric function (>95% accuracy), the super capacity finding in LL but not HH supports the idea that the auditory noise reduction process more effectively cancels the noise at the lower (but high-accuracy) SNRs than at the higher SNRs via a super capacity result.

Because it seems highly likely that our antiphasic effects will appear at other SNRs than those used here (i.e., ours are not “privileged” in any way), these “ceiling-like” SNR effects may be considered as evidence for some type of gain control. That is, it appears that the auditory system uses the differences in signal temporal characteristics to facilitate detection in an SNR-dependent manner. These advantageous interactive mechanisms are not deployed at high SNRs but are only implemented for low SNRs. Although the RTs presented here are on the order of those measured previously (e.g., Kemp, 1984), we must eventually rule out the possibility that the ceiling effects in the HH conditions are not due to a lower limit on the RT.

Future studies will need to be conducted to establish whether the parallel psychometric functions would also be observed in the RT data when using stimuli that do not yield 100% accuracy. Townsend and Altieri (2012) have developed a new capacity metric A(t) which takes into account correct and incorrect trials. This capacity measure will be extremely valuable to determine if these results generalize to SNRs more commonly used in the binaural masking literature, where psychometric functions are measured between chance detection and near-perfect accuracy (Egan et al., 1969; Yasin and Henning, 2012).

Finally, Schröter et al. (2007) argued that super capacity results imply that the two ears are not integrated into a single percept (see also Schröter et al., 2009) and that the redundant signal effect would only occur when the stimuli presented to the two ears do not fuse into a single percept. The results in the *N*_0_*S*_0_ conditions would support this interpretation as we found severely limited capacity when identical stimuli were presented to the two ears. However, the SNR-dependent results in the *N*_0_*S*_π_ conditions do not support such an interpretation in a straightforward way. It seems unlikely that the two ears would be fused into a single percept for the HH, HL, and LH trials but not the LL trials. If anything, one might expect the opposite, as the pure tone would be perceived to “pop out” against the noise background more in the HH conditions (due to the high SNR) than in the LL conditions. However, if the SNR-dependent mechanisms elicit a larger perceptual distinction between the tone and noise at the lower SNRs, it remains possible that tone and noise are perceptually segregated in an SNR-dependent manner. One might speculate that these advantageous mechanisms are employed only when listening is more difficult—there may be no need to implement them in high-SNR situations where detection is essentially trivial.

We conclude by advocating an approach that synthesizes accuracy psychophysics together with response time based information processing methodology. We have demonstrated that RT can be a useful tool for assessment of the binaural system. These results support the idea that a combination of both accuracy and RT methods could be enhance our understanding of perceptual mechanisms in many different modalities.

### Conflict of interest statement

The authors declare that the research was conducted in the absence of any commercial or financial relationships that could be construed as a potential conflict of interest.

## Footnotes

^1^Note that 1/2 of the no trials were binaural trials whereas only 1/3 of the yes trials were binaural. In this case, then there could be a bias toward a “no” response when a binaural noise is heard. Additional data collection suggests that this bias did not lead to a difference in the results presented here.
